# Metabolic determinants of the immune modulatory function of neural stem cells

**DOI:** 10.1186/s12974-016-0667-7

**Published:** 2016-09-02

**Authors:** Denise Drago, Veronica Basso, Edoardo Gaude, Giulio Volpe, Luca Peruzzotti-Jametti, Angela Bachi, Giovanna Musco, Annapaola Andolfo, Christian Frezza, Anna Mondino, Stefano Pluchino

**Affiliations:** 1 CNS Repair Unit, Institute of Experimental Neurology (INSPE), Division of Neurosciences, San Raffaele Scientific Institute, 20132 Milan, Italy; 2 Lymphocyte Activation Unit, San Raffaele Scientific Institute, 20132 Milan, Italy; 3 Medical Research Council Cancer Unit, Hutchison/MRC Research Centre, Cambridge, CB2 0XZ UK; 4 Biomolecular Mass Spectrometry Unit, Division of Genetics and Cell Biology, San Raffaele Scientific Institute, 20132 Milan, Italy; 5 Biomolecular NMR Unit, San Raffaele Scientific Institute, 20132 Milan, Italy; 6 ProMiFa, Protein Microsequencing Facility, San Raffaele Scientific Institute, 20132 Milan, Italy; 7 Department of Clinical Neurosciences, Wellcome Trust–Medical Research Council Stem Cell Institute and National Institute for Health Research Biomedical Research Centre, University of Cambridge, Hills Road, CB2 0HA Cambridge, UK

**Keywords:** Metabolomics, Neural stem cells, Immune modulation, Lymph node cells, Arginase I

## Abstract

**Background:**

Neural stem cells (NSCs) display tissue trophic and immune modulatory therapeutic activities after transplantation in central nervous system disorders. The intercellular interplay between stem cells and target immune cells is increased in NSCs exposed to inflammatory cues. Here, we hypothesize that inflammatory cytokine signalling leads to metabolic reprogramming of NSCs regulating some of their immune modulatory effects.

**Methods:**

NSC lines were prepared from the subventricular zone (SVZ) of 7–12-week-old mice. Whole secretome-based screening and analysis of intracellular small metabolites was performed in NSCs exposed to cocktails of either Th1-like (IFN-γ, 500 U/ml; TNF-α, 200 U/ml; IL-1β, 100 U/ml) or Th2-like (IL-4, IL-5 and IL-13; 10 ng/ml) inflammatory cytokines for 16 h in vitro. Isotopologues distribution of arginine and downstream metabolites was assessed by liquid chromatography/mass spectrometry in NSCs incubated with U-^13^C_6_ L-arginine in the presence or absence of Th1 or Th2 cocktails (Th1 NSCs or Th2 NSCs). The expression of arginase I and II was investigated in vitro in Th1 NSCs and Th2 NSCs and in vivo in the SVZ of mice with experimental autoimmune encephalomyelitis, as prototypical model of Th1 cell-driven brain inflammatory disease. The effects of the inflammatory cytokine signalling were studied in NSC-lymph node cells (LNC) co-cultures by flow cytometry-based analysis of cell proliferation following pan-arginase inhibition with N^ω^-hydroxy-nor-arginine (nor-NOHA).

**Results:**

Cytokine-primed NSCs showed significantly higher anti-proliferative effect in co-cultures vs. control NSCs. Metabolomic analysis of intracellular metabolites revealed alteration of arginine metabolism and increased extracellular arginase I activity in cytokine-primed NSCs. Arginase inhibition by nor-NOHA partly rescued the anti-proliferative effects of cytokine-primed NSCs.

**Conclusions:**

Our work underlines the use of metabolic profiling as hypothesis-generating tools that helps unravelling how stem cell-mediated mechanisms of tissue restoration become affected by local inflammatory responses. Among different therapeutic candidates, we identify arginase signalling as novel metabolic determinant of the NSC-to-immune system communication.

**Electronic supplementary material:**

The online version of this article (doi:10.1186/s12974-016-0667-7) contains supplementary material, which is available to authorized users.

## Background

Compelling evidence is available that transplanted stem cells promote significant clinico-pathological recovery in several preclinical models of neurological disorders such as multiple sclerosis, cerebral stroke and spinal cord injury [1–4].

Remarkable neuroprotective and immune modulatory capacities have been identified for neural stem cells (NSCs) that may result from consistent mechanisms of intercellular interplay with target host cells and may predominantly account for the therapeutic effects of NSC grafts [2, 5–9]. The putative mechanisms that sustain both repair capabilities, as well as long-term functional integration of NSCs upon transplantation, are yet to be fully understood.

Rodent NSCs inhibit the activation and proliferation of antigen-specific and antigen non-specific Th1 and Th17 cells in vitro, as well as promote T cell apoptosis [6, 9–11]. Human NSCs suppress the proliferation and alter the profile of cytokine secretion of xenogeneic antigen-specific and allogeneic mitogen-activated T cells [12, 13]. Compared to rodent NSCs, human NSCs have a lower cytotoxicity towards T cells but a higher cytotoxicity towards monocyte/macrophages in vitro [14]. Human NSCs also hinder the differentiation of myeloid precursor cells (MPCs) into immature dendritic cells (DCs), and the maturation of immature DCs to functional antigen-presenting cells [13]. Recent studies have also shed new light on the understanding of mechanisms of transcellular information exchange and demonstrated that cytokine-regulated exosome signalling is an important pathway by which NSCs exploit some of their protective capacities [15]. In parallel to these functional studies, the investigation of the *stem cell secretome* has gained increasing attention in recent years because of its multiple implications for the potential reparative, restorative, or regenerative applications of stem cell medicines [16–19]. Paracrine signalling mediated by stem cells plays an essential role in the reparative process observed after stem cells transplantation, with stem cells secreting growth factors, chemokines and cytokines, both constitutively as well as in response to priming with pro-inflammatory molecules [17, 18, 20–23].

Thus, the concept that stem cells solely act as directly repairing cells is now being revisited and enriched with the emerging view that stem cells secrete certain regenerative factors in response to environmental stimuli, which include cytokines, growth factors, morphogens and toll-like receptor (TLR) ligands [16, 24]. Hypoxic preconditioning, exposure to inflammatory cytokines or mechanical and shear stress conditioning (e.g. growing cells in 3D spheres or scaffolds) have all been shown to promote the release of different potential therapeutic small molecules [24, 25].

The ability of stem cells to secrete neuroprotective and immune modulatory factors indicates that there is still a lot to learn about functional stem cell plasticity, especially when the regulation of host responses is enhanced after licensing or priming with inflammatory cytokines such as for NSCs [21].

Metabolomics is a promising complementary approach to explore the functional stem cell response to cellular signalling and is defined as the metabolic complement of functional genomics. Metabolomics enables the systematic analysis of small metabolites involved in biochemical reactions, revealing connections between different pathways that operate within living cells [26–30]. The identity, concentration and fluxes of metabolites are the final product of interactions between gene expression, protein expression and the cellular environment. Thus, metabolomics amplifies changes both in the proteome and the genome and represents a more accurate approximation to the phenotype of an organism in health and disease [31, 32].

We exploited metabolomics to investigate whether cytokine signalling leads to metabolic reprogramming of NSCs driving some of their immune modulatory effects.

To this aim, we sought to measure small molecules from undifferentiated mouse NSCs and anticipated that these compounds were altered in NSCs primed with inflammatory cytokines. Whole secretome-based screening and analysis of intracellular small metabolites were performed in NSCs after exposure to a cocktail of Th1-like or Th2-like inflammatory cytokines as in vitro system mimicking the putative inflammatory niche that has been described to induce an immune modulatory phenotype in stem cells in vivo [3].

Our high-throughput *omic* approach defined the arginine metabolism to be mostly altered in Th1 NSCs. In parallel, we found that NSCs constitutively expressed both intracellular arginase II and extracellular arginase I, while arginase inhibition by N^ω^-hydroxy-nor-arginine (nor-NOHA) blocked some of the immune modulatory effects of Th1 NSCs.

Our work underlines the use of the NSC metabolome as a hypothesis-generating tool for the identification of candidate biomarkers that will predict or measure pharmacological efficacy or toxic responses. It also identifies arginase signalling as a novel metabolic determinant of the NSC-to-immune system communication to be exploited in the manipulation of the NSC activities in therapeutic settings.

## Methods

### NSC preparations

NSCs lines were prepared from the subventricular zone (SVZ) of 7–12-week-old female SJL mice (18–20 gr. Charles River). NSCs were grown in a serum-free basal medium, NeuroCult basal medium plus mouse NeuroCult proliferation supplements (Complete Growth Medium, CGM; Stem Cell Technologies) supplemented with 2 μg/ml heparin (Sigma-Aldrich), 20 ng/ml purified human recombinant epidermal growth factor (EGF; Provitro) and 10 ng/ml human recombinant fibroblast growth factor (FGF)-2 (Provitro) [9]. At time of passaging (every 3–5 days), neurospheres were dissociated by enzymatic digestion with Accumax (Sigma) at 37 °C for 15 min, the number of viable cells determined by trypan blue exclusion and viable cells re-plated at 8000 cells/cm^2^ (1.3 × 10^6^ cells with 15-ml media in T162 flask) [33]. NSCs at passage number ≤15 were used in all experiments.

### Th1 and Th2 cytokine priming

NSCs were plated in CGM with or without either Th1-like (200 U/ml recombinant mouse TNF-α, Pepro Tech Inc.; 500 U/ml recombinant mouse IFN-γ, Pepro Tech Inc; 100 U/ml recombinant mouse IL-1β, Pepro Tech Inc) or Th2-like (10 ng/ml recombinant murine IL-4, R&D; 10 ng/ml recombinant mouse IL-5, R&D; 10 ng/ml recombinant mouse IL-13, R&D) cytokine cocktails for 16 h in vitro [33]. At the end of the conditioning, NSCs were washed three times with phosphate-buffered saline (PBS) to remove cytokine contamination before cell harvesting. Finally, NSCs and NSC-conditioned media were processed according to the analysis to be performed.

### Metabolomic analysis by liquid chromatography/mass spectrometry (LC/MS) and gas chromatography/mass spectrometry (GC/MS)

#### Sample preparation

NSCs were plated at 348,000 cells/ml (5.2 × 10^6^ cells with 15-ml media in T162 flask) in CGM added with either Th1-like or Th2-like cytokine cocktails as above. Basal, Th1, and Th2 NSCs were harvested (10^7^ cells per sample), and the supernatants immediately collected to be analysed by using Metabolon platform service. Five biological replicates of NSCs pellets were washed twice with PBS and then snap-frozen on dry ice and stored at −80 °C until shipment to Metabolon. The sample preparation process was carried out using the automated MicroLab STAR® system from Hamilton Company. Recovery standards were added prior to the first step in the extraction process for quality control (QC) purposes. Sample preparation was conducted using a proprietary series of organic and aqueous extractions to remove the protein fraction while allowing maximum recovery of small molecules. The resulting extract was divided into two fractions: one for analysis by LC and one for analysis by GC. The samples were placed briefly on a TurboVap® (Zymark) to remove the organic solvent. Each sample was then frozen and dried under vacuum. The samples were then prepared for the appropriate instrument, either LC/MS or GC/MS.

#### Quality assurance (QA)/quality control (QC)

For quality assurance QA/QC purposes, additional samples were included with each day’s analysis. These samples included a well-characterized pool of human plasma, a pool of a small aliquot of each experimental sample, an ultra-pure water process blank and an aliquot of solvents used in extraction to segregate contamination sources in the extraction. Furthermore, a selection of QC compounds was added to every sample, including those under test. These compounds were chosen carefully so as not to interfere with the measurement of the endogenous compounds.

#### GC/MS

The samples destined for GC/MS analysis were re-dried under vacuum desiccation for a minimum of 24 h prior to being derived under dried nitrogen using bistrimethyl-silyl-triflouroacetamide (BSTFA). The GC column was 5 % phenyl and the temperature ramp was from 40 to 300 °C in a 16-min period. The samples were analysed on a Thermo-Finnigan Trace DSQ fast-scanning single-quadrupole mass spectrometer using electron impact ionization. The instrument was tuned and calibrated for mass resolution and mass accuracy on a daily basis. The information output from the raw data files was automatically extracted as discussed below.

#### Accurate mass determination and MS/MS fragmentation (LC/MS), (LC/MS/MS)

The LC/MS portion of the platform was based on a Waters ACQUITY UPLC and a Thermo-Finnigan LTQ-FT mass spectrometer, which had a linear ion trap (LIT) front end and a Fourier transform ion cyclotron resonance (FT-ICR) mass spectrometer back end. For ions with counts greater than two million, an accurate mass measurement could be performed. Accurate mass measurements could be made on the parent ion as well as fragments. The typical mass error was less than 5 ppm. Ions with less than two million counts require a greater amount of effort to characterize. Fragmentation spectra (MS/MS) were typically generated in a data-dependent manner, but if necessary, targeted MS/MS could be employed, such as in the case of lower level signals.

### Western blotting

NSCs were plated at 348,000 cells/ml, collected after 16 h of incubation in CGM without or with Th1 or Th2 cytokines and washed with PBS. Conditioned media were collected and immediately frozen at −80 °C before the protein quantification. Protein quantification was performed using Direct Detect Spectrometer (Millipore). One hundred microgram of total protein for NSC-conditioned media were separated by SDS-PAGE using 4–12 % precast NuPAGE Bis-Tris gels (1.5-mm thickness) under reducing (Reducing Buffer, 10×) conditions and MES running buffer and then transferred onto nitrocellulose membranes (0.45-μm pore size, Hybond ECL) using XCell II Blot Module and NuPAGE transfer buffer (all from Invitrogen). Molecular weight marker: SeeBluePlus2PrestainedStandard (Invitrogen). For immunoblot analysis, the membranes were blocked for 1 h at room temperature with Tris-buffered saline/Tween (TBST; 10 mM Tris-HCl, 150 mM NaCl, 0.1 % Tween 20 pH 7.6) containing 5 % nonfat dry milk and then incubated overnight at 4 °C with rabbit anti-arginase I (H-52, Santa Cruz Biotech) or anti-arginase II antibody (H-64, Santa Cruz Biotech) by using antibody dilution 1:200. Mouse liver (sc-2256, Santa Cruz) and rat kidney (sc-2394, Santa Cruz Biotech) extracts were used as positive control for arginase I and arginase II, respectively. After washing with TBST, the nitrocellulose membranes were incubated with the appropriate donkey anti rabbit IgG HRP antibody (Amersham NA9340V; dilution used: 1:3000) for 1 h at room temperature. Immunoreactivity was revealed by using an ECL detection kit (Pierce).

### Measurement of ^13^C-labelled metabolites by LC/MS

1 ×10^6^ cells were plated in T25 flasks and cultured in CGM supplemented with U-^13^C_6_ L-arginine (Cambridge Isotopes Laboratories) for 2 h and 16 h in the absence and in the presence of Th1 and Th2 cytokines cocktails. The cells were then harvested, washed with PBS (2 × 5 min), and lysed for 15 min at 4 °C under agitation with 1 ml of 50 % methanol and 30 % acetonitrile in water kept in dry ice/methanol bath (−80 °C). The insoluble material was pelleted in a cooled centrifuge at 14,000*g* for 10 min at 4 °C and the supernatant were collected for LC-MS analysis. XBridge Amide column (3.5 μm, 150 × 2.1 mm) was used for LC separation and the detection of metabolites was performed using a Thermo Scientific Q-Exactive high-resolution mass spectrometer with electrospray (ESI) ionization, examining metabolites in both positive and negative ion modes, over the mass range of 75–1000 m/z. The mobile phase for elution was a gradient established between water acidified with 0.1 % formic acid (A) and acetonitrile acidified with 0.1 % formic acid (B) at a flow rate of 200 μl/min. The gradient used was preceded by an isocratic step at 20 % A/80 % B. This step was followed by a linear decrease of B at 20 % in 25 min and re-equilibration at 20 % A/ 80 % B for 11 min. The injected volume was 5 μl.

### Urea cycle colorimetric assay

1.74 × 10^6^ cells (cellular density 348,000 cells/ml) were seeded onto T25 flask (5 ml medium) in the absence or in the presence of either Th1 or Th2 cytokines cocktails. NSCs at different experimental conditions were homogenized in 100 μl Assay Buffer (Urea Assay Colorimetric Kit, Biovision), centrifuged at 15,000*g* for 10 min to remove insoluble materials. Fifteen-microliter samples were directly added to a 96-well plate for the urea determination according to the manufacturer’s instructions.

### Arginase enzymatic activity

The enzymatic activity of arginase within NSCs (with or without Th1 or Th2 cytokines cocktails) was determined by arginase activity colorimetric assay kit (BioVision). Briefly, 1 × 10^6^ cells at the same cellular density used for the metabolomic studies (348,000 cells/ml) were dissolved with 100 μl ice-cold arginase buffer and centrifuged at 10,000*g* for 5 min. The supernatants were collected, and 40 μl were used to perform the assay for each samples. 

### NSC/lymph node cell co-culture

Axillary, brachial, cervical, inguinal, and mesenteric lymph nodes were surgically excised from SJL mice and reduced to single cell suspension by mechanical disruption.

In selected experiments, CD4^+^ T cells were purified by negative selection using anti-CD8 (clone KT1.5) and anti-I-Ab-d/I-E (clone B21-22) rat Abs and sheep anti-rat-coated magnetic beads (Dynal Biotech, UK) to a purity of 95 %. Purified CD4 lymphocytes or unfractionated lymph node cells (LNC) were seeded in 96-well plates (1 × 10^5^ per well) in a final volume of 200 μl of CGM and were stimulated with anti-mouse-CD3/CD28 beads (Dynabeads mouse T-activator CD3/CD28, Invitrogen) at a ratio of 1:1 or left untreated. NSCs were pre-conditioned for 16 h with Th1 or Th2 cytokines cocktails at 348,000 cells/ml and then co-cultured with LNCs (NSC/LNC ratio 1:2) in the same well. NSCs were washed three times with PBS to avoid any cytokine carry-over in the co-culture system.

In selected experiments, LNCs were labelled with the vital dye, 5-(and-6)-carboxyfluorescein diacetate succinimidyl ester (CFSE; Molecular Probes, Invitrogen), final concentration of 1 μmol/L. Briefly, the cells were washed twice with PBS and suspended at 20 × 10^6^/ml in PBS. An equal volume of CFSE at 2 μmol/L was then added. After 8-min incubation under gentle shaking at room temperature, an equal volume of FCS was added to quench the reaction. LNCs were then washed twice in RPMI with 10 % FCS and plated with NSCs in co-cultures for 48 and 72 h, as above. LNCs were surface stained with rat anti-mouse CD4 Pacific Blue conjugated (clone RM4-5, BD Pharmingen) and CD44PerCP-Cy5.5 (BioLegend). The nuclear counterstain and dead cell indicator TO-PRO3 Iodide (Molecular Probes) was added before the flow cytometry acquisition. In a parallel co-culture experiment, the cells were labelled for the last 5 h of a 72-h-long culture with 10 μM 5-ethynyl-2′deoxyuridine (EdU). EdU-Click iT Flow Cytometry Assay (Alexa 647, Invitrogen, Molecular Probes) was used to detect EdU incorporation according to the manufacturer’s instructions (reaction volumes were adjusted to 50 μl). LNCs were surface stained with rat anti-mouse CD4 Pacific Blue conjugated (clone RM4-5) and CD44PerCP-Cy5.5.

N^ω^-hydroxy-L-arginine (nor-NOHA; 30 μM) was added to cultures to inhibit arginase activity. Flow cytometry was performed with FACSCalibur (BD Biosciences), and a minimum of 15,000 events were acquired. Analysis of FACS data was performed using the FlowJo_V10 software.

### Arginase I and II expression on NSCs in vitro

Analysis of basal and cytokine-induced expression and localization (cytosol vs. mitochondria) of arginase I and II was done on mouse NSCs transduced with a 3rd generation Mito-DsRed lentiviral vector. NSCs were plated in CGM (laminin 1:100) at a density of 80,000 cells/cm^2^. After 12 h, the cells were treated for additional 16 h in vitro with Th1 or Th2 cytokine cocktails as above. Untreated NSCs were used as controls. NSCs were fixed with paraformaldehyde (PFA) 4 % and then stained using a chicken anti-nestin (1:500; Abcam) and a goat anti-arginase I (1:100; Santa Cruz) or a rabbit anti-arginase II (1:100; Santa Cruz) primary antibodies. Appropriate Alexa Fluor conjugated secondary antibodies were then used. Nuclei were counterstained with 4, 6-diamidino-2-phenylindole (DAPI). For the assessment of arginase I and II expression, immunofluorescent staining were evaluated adopting a CCD camera/fluorescent microscope; *n* = 5 equally distributed regions of interest (ROIs) were chosen and acquired via ×20 objective lens for analysis (*n* = 4 cover slides were used for each group). Fluorescence intensity was calculated with ImageJ Software (NIH) and normalized on the number of DAPI^+^ cells/ROI. Data were expressed as arginase I or II mean normalized intensities over micron^2^ ± SEM.

### Arginase I and II expression on NSCs in vivo

Ex vivo histopathological fluorescent images of ArgI/ArgII expression at level of SVZ were obtained from 4–8-week-old C57BL/6 female mice with myelin oligodendrocyte glycoprotein (MOG) peptide 35–55 induced experimental autoimmune encephalomyelitis (EAE) at 25 and 50 days post immunization (dpi), as described [33]. Strain-, age-, sex- and weight-matched healthy mice were used as controls. The mice were transcardially perfused with saline EDTA and 4 % paraformaldehyde. Brains were removed and post-fixed in PFA 4 % for 24 h at 4 °C and then washed with PBS. The brains were cryoprotected with 30 % sucrose (Sigma) and then embedded in optimal cutting temperature (OCT) Tissue Tek compound (EM sciences) for freezing. Twenty five-micrometer-thick coronal brain sections were cut and collected onto SuperfrostPlus slides (Menzel-Glaser, Thermo Fisher Scientific) and processed for histopathology. The following primary antibodies were used: goat anti-arginase I (1:100 Santa Cruz), rabbit anti-arginase II (1:100 Santa Cruz), chicken anti-nestin (1:500 Abcam) and a rat anti-CD45 (BD). Appropriate Alexa Fluor-conjugated secondary antibodies were used. Nuclei were counterstained with DAPI. Confocal microscopy images of SVZ healthy controls and MOG-induced EAE mice were acquired at ×40 and ×63.

### Statistical analysis

Statistically significant differences between experimental groups were determined by Kruskal-Wallis followed by unpaired two-tailed *t* test (for comparisons between groups). */°/^*p* ≤ 0.05; **/°°*p* ≤ 0.01; ***p ≤ 0.001 and n.s. = not significant. Statistical analyses were performed using Prism software (v6.0f, GraphPad Software, San Diego, CA).

## Results

### In vitro NSCs priming with inflammatory cytokines potentiates their anti-proliferative effect on T cells

Priming with environmental stimuli that include cytokines, growth factors, morphogens and toll-like receptor (TLR) ligands modulates several stem cell functions [16, 34]. Here, we asked whether exposing NSCs to inflammatory cytokines, that are known to play a central role in triggering and perpetuating chronic inflammation, induces a *licensing switch* in their intrinsic immune regulatory functions [35].

To evaluate the effects of NSCs on acute T cell responses, we measured CD3/CD28-induced lymph node cell (LNC) proliferation by flow cytometry using a carboxyfluorescein diacetate succinimidyl ester (CFSE) dilution assay at both 48 and 72 h in vitro. These time points were selected based on previous data on proliferation and activation of rodent LNCs in vitro [36]. LNCs were also stained with anti-CD44 antibodies to detect their activation. NSCs were first primed for 16 h in vitro with a Th1-like cytokine cocktail composed of IFN-γ, TNF-α and IL-1β or a Th2-like cytokine cocktail composed of IL-4, IL-5 and IL-13 [33], and then tested for their immune modulatory activity in same well co-cultures with LNCs (Th1 NSC/LNC and Th2 NSC/LNC, respectively). Co-cultures of basal (e.g. not exposed to cytokines) NSCs and LNCs (NSC/LNC) or LNC preparations only (LNC) were used as controls.

**Fig. 1:**
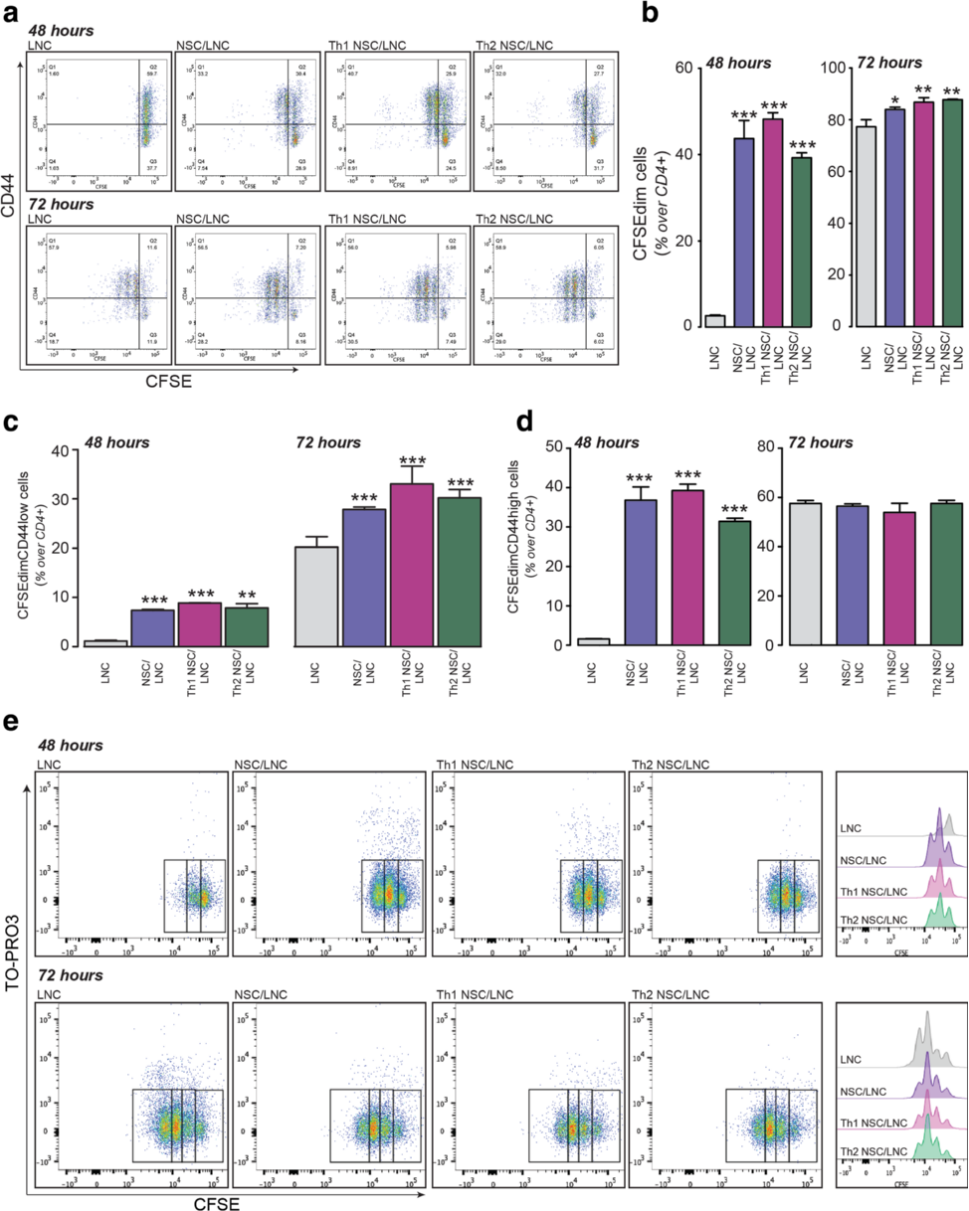
Cytokine-primed NSCs hinder lymph node cell (LNC) proliferation and cause premature CD44 downregulation. Unfractionated LNC were labelled with the vital dye CFSE, cultured on CD3/CD28 coated mAb in the absence or the presence of NSCs, Th1 NSCs, or Th2 NSCs for 48 and 72 h in vitro. **a** Cells were surface stained with anti-CD4 and CD44 antibodies. *Dot plots* depict relative CD44 and CFSE levels and are representative of gated CD4^+^ viable cells. **b–d** Absolute fractions of CFSE^dim^ (**b**), CFSE^dim^CD44^low^ (**c**), and CFSE^dim^CD44^high^ CD4^+^ T cells are shown (**d**). Data in **b–d** are expressed as mean % (±SD) and have been collected out of *n* ≥ 3 independent experiments. **e** Representative dot plots of relative CFSE and TO-PRO3 levels after gating on CD4^+^ T cells as in **a–d**. Unpaired two-tailed *t* test for comparisons between groups were applied; *p ≤ 0.05; **p ≤ 0.01; ***p ≤ 0.001 vs. LNC

By 48 h, significantly higher proportions of CD4^+^ T cells started to proliferate (CSFE^dim^) in NSC/LNC [43.7 % (±4.2)], Th1 NSC/LNC [48.2 % (±1.5)] and Th2 NSC/LNC [39.2 % (±1.1)] vs. LNC [2.64 % (±0.18); *p* ≤ 0.0001] (Fig. 1a, b). By 72 h, the fraction of CFSE^dim^ CD4^+^ T cells further increased in all conditions, still being slightly more represented in NSC/LNC [83.9 % (±0.9)], Th1 NSC/LNC [86.8 % (±1.7)] and Th2 NSC/LNC [87.8 % (±0.1)], vs. LNC [77.3 % (±2.72); *p* ≤ 0.05 and *p* ≤ 0.01] (Fig. 1a, b). Nevertheless, we noticed a significantly higher proportion of CFSE^dim^ CD4^+^ T cells expressing low levels of the activation marker CD44 (CFSE^dim^CD44^low^CD4^+^) within NSC/LNC, Th1 NSC/LNC and Th2 NSC/LNC at both 48 and 72 h [*p* ≤ 0.001 vs. LNC] (Fig. 1a, c). Comparable results were obtained when NSCs were co-cultured with purified CD4^+^ T cells for 72 h in vitro (Additional file 1: Figure S1), thus suggesting a direct effect of NSCs on CD4^+^ T cells.

Significantly higher proportions of CSFE^dim^CD44^high^ CD4^+^ T cells were also observed by 48 h in NSC/LNC [36.8 % (±3.3)], Th1 NSC/LNC [39.3 % (±1.6)], and Th2 NSC/LNC [31.4 % (±0.8)] compared to LNC [1.6 % (±0.1); *p* ≤ 0.0001]. By contrast, no differences in the proportion of CD4^+^CSFE^dim^CD44^high^ T cells were observed by 72 h in any of the conditions tested (Fig. 1a, d).

LNCs in co-cultures at 72 h also showed a higher CFSE content (CFSE^high^) vs. control LNC (Fig. 1e). This CFSE^high^ late LNC behaviour did not account for any significant differences in the proportion of TO-PRO3^+^ apoptotic CD4^+^ T cells; yet, it was associated to significant 4–8-fold higher survival of LNCs in co-cultures [*p* ≤ 0.01 and *p* ≤ 0.001] vs. control LNCs (Additional file 2: Figure S2).

We therefore reasoned that these results might suggest that only in the presence of NSCs, T cells survive better and undergo transient early activation and proliferation, while prematurely exiting from the cell cycle [37]. To address this hypothesis, we analysed the ability of T cells in co-cultures to enter S-phase (synthesis phase) of the cell cycle at 72 h upon exposure to a 5-h-long pulse with 5-ethynyl-2′-deoxyuridine (EdU).

**Fig. 2:**
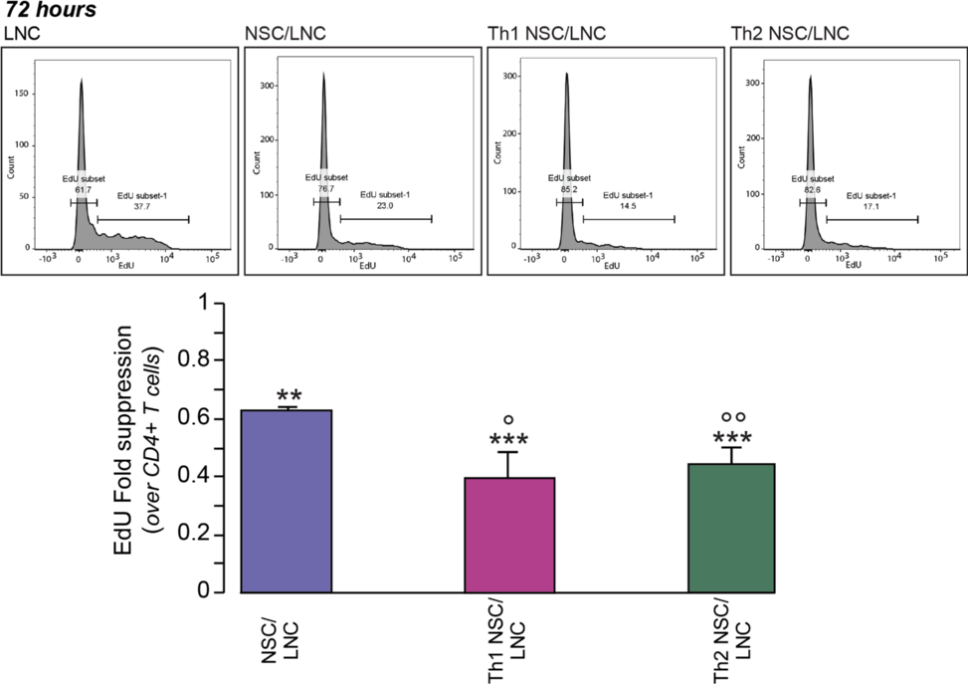
Cytokine-primed NSCs hinder cell cycle progression in LNC in vitro. Unfractionated LNC were co-cultured with NSC for 72 h as in Fig. 1. EdU was added to the cells for the last 5 h of culture. Cells were then recovered, labelled with anti-CD4 mAb, stained for intracellular EdU content, and analysed by flow cytometry. Events are depicted after gating on CD4^+^ T cells. Relative fractions of EdU^+^ cells are represented. Data are expressed as mean EdU fold suppression (±SD) calculated by dividing the percent of EdU^+^CD44^high^ cells over the control CD4^+^ T cells from *n* ≥ 3 independent experiments. Unpaired two-tailed *t* test for comparisons between groups were applied; **p ≤ 0.01 and ***p ≤ 0.001 vs. LNC; °*p* ≤ 0.05 and °°*p* ≤ 0.01 vs. NSCs/LNC

We found a significant 38 % reduction of the fraction of CD4^+^ T cells incorporating EdU in NSC/LNC co-cultures [22.50 % (±0.4)] vs. control LNC [35.80 % (±2.9); *p* ≤ 0.01] (Fig. 2). This effect became even more striking in Th1 NSC/LNC and Th2 NSC/LNC, which showed 56–61 % inhibition of the EdU uptake [Th1 NSC/LNC: 14.17 % (±3.3); Th2 NSC/LNC: 15.9 % (±1.8); all *p* ≤ 0.001 vs. controls], thus confirming that cytokine-induced NSC-reactive signals may hinder optimal cell cycle progression in target T cells [8].

Together, these data confirm the ability of NSCs to affect T cell activation and proliferation and suggest that cytokine signalling (*priming*) on NSCs provides a higher anti-proliferative effect.

### Untargeted metabolomic studies identifies defective arginine metabolism in Th1 NSCs

To investigate whether the exposure to a Th1-like cytokine cocktail induced a metabolic alteration in NSCs, which could interfere with the NSCs-T cell crosstalk, we performed untargeted intracellular (*endometabolome*) and extracellular (*exometabolome*) metabolomic profiling of basal and Th1 NSCs. NSCs primed with Th2-like cytokine cocktails (Th2 NSCs) were used as additional controls. GC/MS and LC/MS in positive ion mode and negative ion mode were performed according to the different metabolite ionization properties. In total, 215 and 86 metabolites were identified for the NSCs *endo* and *exo*, when comparing basal, Th1 or Th2 NSCs. These analytes spanned many classes of metabolites, including amino acids, carbohydrates, peptides, lipids and nucleotides (Additional file 3: Table S1).

Welch’s two-sample *t* tests were used to identify *exo-* and *endo-*metabolites that differed significantly between the experimental groups (Tables 1 and 2). On the one hand, we found a total of 14 and 7 *exo*-metabolites that were significantly upregulated in the conditioned media of Th2 NSCs and Th1 NSCs, respectively, compared to basal NSCs. On the other hand, we found a total of 2 and 1 *exo*-metabolites that were significantly downregulated in the conditioned media of Th2 NSCs and Th1 NSCs, respectively, compared to basal NSCs (Table 1).

**Table 1:**
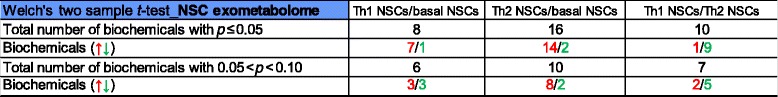
Summary table for untargeted endo and exo-metabolomic NSC profiling: NSC exometabolome

Welch’s two-sample *t* tests were used to identify metabolites that are significantly regulated between experimental groups within cells (Table 1) or in the media (Table 2) (up regulated metabolites are in red whereas downregulated metabolites are in green)

**Table 2:**
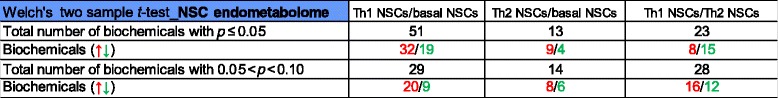
Summary table for untargeted endo and exo-metabolomic NSC profiling: NSC endometabolome

Welch’s two-sample *t* tests were used to identify metabolites that are significantly regulated between experimental groups within cells (Table 1) or in the media (Table 2) (upregulated metabolites are in red whereas downregulated metabolites are in green)

When we instead analysed the *endometabolome* of NSCs, we found that Th1 NSCs had more pronounced alterations with respect to Th2 NSCs and basal NSCs. On the one hand, a total of 32 endo-metabolites were significantly upregulated in Th1 NSCs (*p* ≤ 0.05 vs. basal NSCs) compared to the only 9 endo-metabolites that were significantly upregulated in Th2 NSCs. On the other hand, 19 endo-metabolites were significantly downregulated in Th1 NSCs (*p* ≤ 0.05 vs. basal NSCs) compared to the only 4 endo-metabolites downregulated in Th2 NSCs (Table 2).

**Fig. 3:**
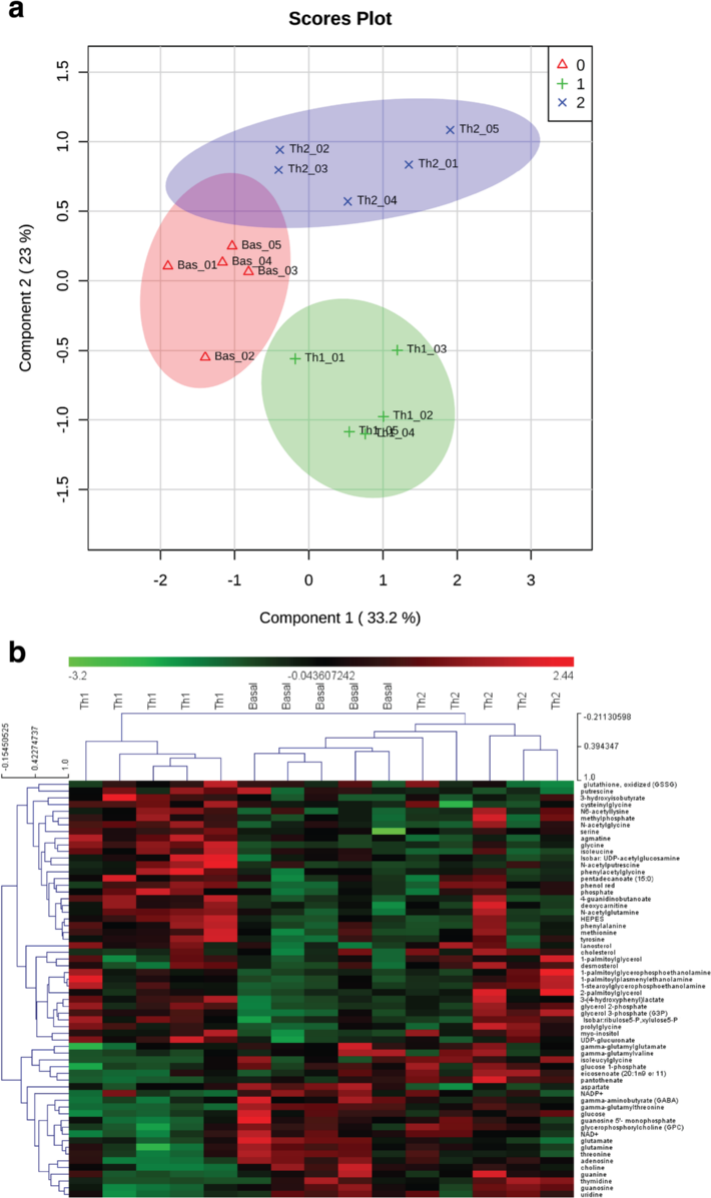
Untargeted metabolomics analysis of NSCs under Th1- and Th2-like cytokines: Multivariate analysis (PLS-DA) and hierarchical clustering. **a** PLS-DA score assessing the clustering of the three sample groups (basal NSCs (group 0), Th1 NSCs (group 1), or Th2 NSCs (group 2)) was performed on significant metabolites by using MetaboAnalyst 2.0 software. **b** Hierarchical clustering heatmap for MS peak intensities of significantly (*p* ≤ 0.05) different intracellular metabolites among basal NSCs, Th1 NSCs, or Th2 NSCs. MultiExperiment Viewer software (MeV) (v 4.6.2) was applied on NSCs significant endometabolome dataset with Pearson correlation metric. Sixty-one intracellular metabolites were significantly different in the three experimental conditions among 215 identified metabolites by LC-MS/MS and GC-MS/MS

Most importantly, partial least squares discriminant analysis (PLS-DA) clearly segregated basal, Th1 or Th2 NSCs in separate clusters (Fig. 3a). Each condition clustered separately, thus indicating that each NSC metabolic profiles were highly specific. Out of the 215 identified endo-metabolites, 61 were significantly different between basal, Th1, and Th2 NSCs, as depicted in the heatmap of the hierarchical clustering of the MS peak intensities (Fig. 3b; *p* ≤ 0.05).

**Fig. 4:**
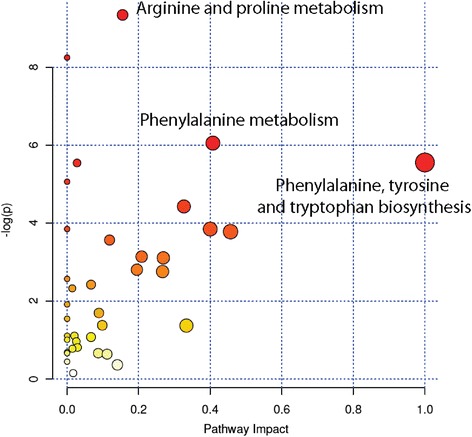
Metabolic pathways analysis of Th1 NSCs. Enriched metabolic pathways were ranked according to their FDR values calculated by the MetPa method implemented in MetaboAnalyst 2.0 software. The most significant pathways for Th1 NSCs compared to basal NSCs were represented by both the *bigger/red dots* and by those *dots with higher log p value*. The pathway impact is calculated as the sum of the importance measures of the matched metabolites normalized by the sum of the importance measures of all metabolites in each pathway

**Fig. 5:**
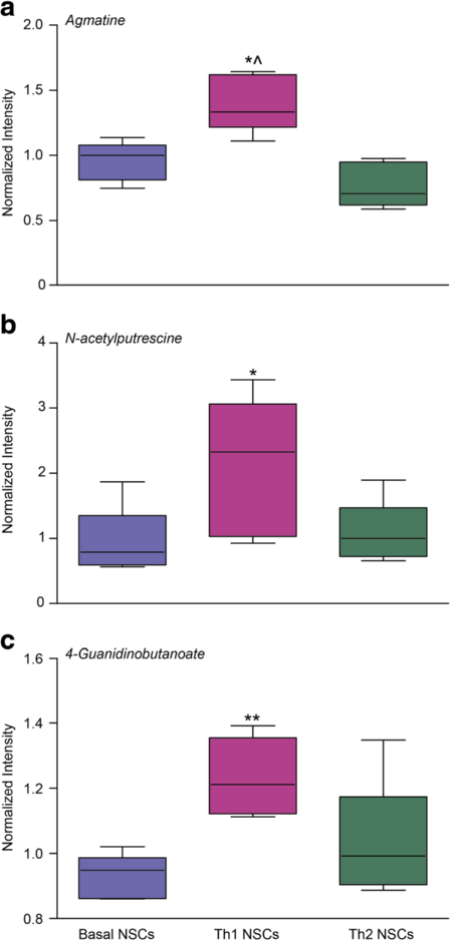
Th1-like cytokines affect NSC arginine metabolism altering the levels of arginine-derived polyamines. *Box plot* of NSCs endo-metabolites for the arginine pathway detected by LC-MS/MS expressed as mean of MS peak intensity for five independent experiments. Agmatine (**a**), N-acetylputrescine (**b**), and 4-guanidinobutanoate (**c**) levels were upregulated in Th1 NSCs compared to basal NSCs. Unpaired two-tailed *t* test for comparisons between groups were applied. *p ≤ 0.05 and **p ≤ 0.01 vs. basal NSCs, ^*p* ≤ 0.05 vs. Th2 NSCs

We then focused on the endometabolome, to investigate the possible metabolic determinants of the enhanced immune regulatory function of Th1 NSC on T cells. To identify the intracellular metabolic pathways that were mostly altered in Th1 NSCs (vs. basal NSCs), we performed a metabolic pathway analysis (MetPA) on differentially represented metabolites by using Metaboanalyst, a web-based tool for metabolomic data interpretations [38]. This analysis identified multiple intracellular metabolic pathways as being altered in Th1 NSCs (vs. basal NSCs). Metabolites belonging to the metabolism of arginine and proline, of phenylalanine and of tyrosine and tryptophan were differentially enriched in Th1 NSCs (vs. basal NSCs) (Fig. 4). In particular, when considering the log *p* value, differences in the arginine and proline metabolism of Th1 NSCs were the most significant when compared to basal NSCs (Additional file 4: Table S2). Accordingly, the arginine downstream metabolites agmatine, N-acetylputrescine and 4-guanidinobutanoate—which are all members of the arginine decarboxylase pathway—were significantly upregulated in Th1 NSCs (*p* ≤ 0.01 and *p* ≤ 0.05 vs. basal and Th2 NSCs) (Fig. 5).

Untargeted metabolomics can therefore be efficiently used to identify several differences in the *endo*- and *exometabolome* of primed NSCs. In particular, we found that arginine and proline are the most differentially regulated in the *endometabolome* of Th1 NSCs.

### Targeted metabolomics identifies defective arginase activity within the urea cycle in Th1 NSCs

**Fig. 6:**
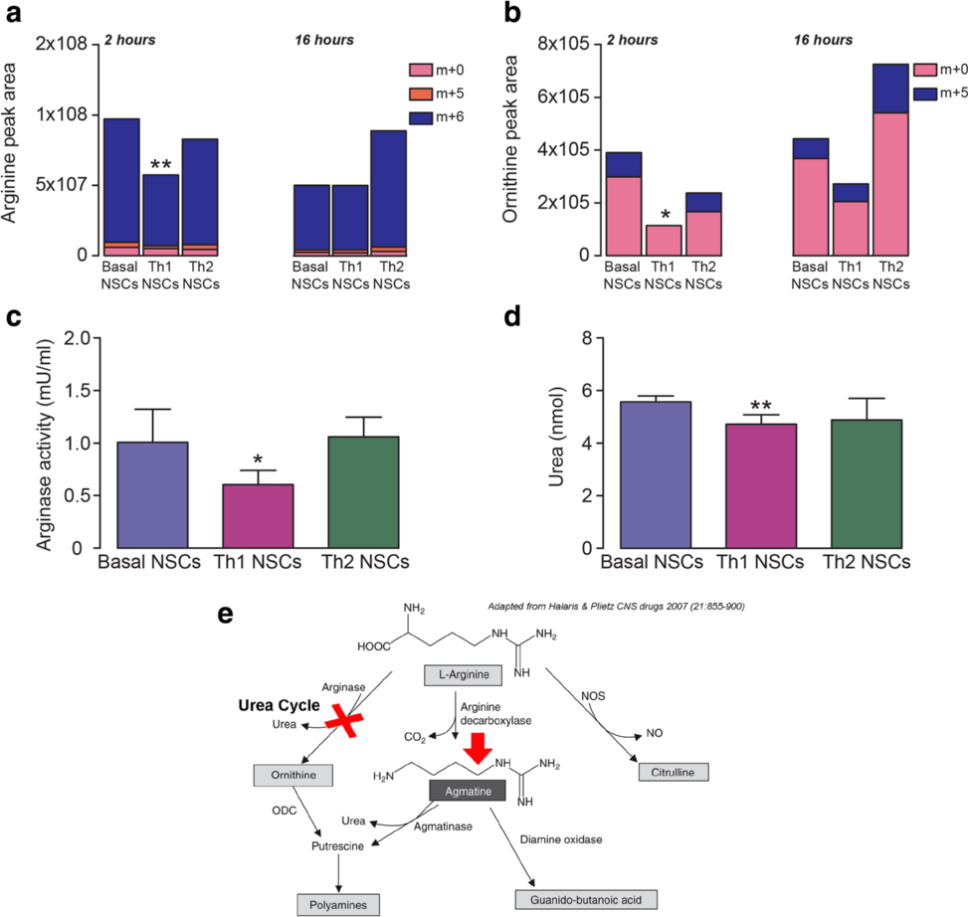
Th1-like cytokines affect NSC arginine metabolism by inducing defective arginase activity within the urea cycle. **a** NSCs were cultured in complete medium supplemented with U-^13^C6 L-arginine for 2 and 16 h in the absence and in the presence of Th1- or Th2-like cytokines. Metabolic flux analysis was performed by measuring all labelled metabolites derived from arginine by LC/MS [labelled arginine (**a**) and ornithine (**b**) herein reported]. **c** Arginase enzymatic activity in basal NSCs as well as Th1 and Th2 NSCs was detected by arginase activity colorimetric assay kit. **d** NSC urea levels were detected by colorimetric assay measuring the ability of NSCs to convert arginine into urea in basal condition and under Th1 or Th2 treatment. **e** Schematic representation of the most significant findings for the metabolic alteration in the NSC arginine metabolism (modified from [59]). *p ≤ 0.05 or **p ≤ 0.01 vs. basal and Th2 NSCs

Arginine levels have been reported to be critical for T cell activation and proliferation [39]. Therefore, we decided to focus on the intracellular arginine metabolism to clarify which metabolic routes were specifically altered in Th1 NSCs. The cells were incubated with uniformly labelled U-^13^C_6_ L-arginine in the absence or in the presence of Th1 and Th2 cytokines and isotopologues distribution of arginine and downstream metabolites was assessed by LC/MS (Fig. 6). Within 2 h, the amount of labelled intracellular arginine (m + 6) was significantly lower in Th1 NSCs (*p* ≤ 0.01), when compared to both basal and Th2 NSCs (Fig. 6a). In addition, labelled ornithine (m + 5) was undetectable in Th1 NSCs by 2 h (Fig. 6b), and overall levels of unlabelled ornithine (m + 0) were lower in these cells (*p* ≤ 0.05). By 16 h, differences in the arginine and ornithine intracellular levels between basal NSCs and Th1 NSCs were no longer statistically significant.

The reduced amount of m + 6 arginine (Fig. 6a) and m + 5 ornithine (Fig. 6b), together with lower unlabelled m + 0 intracellular ornithine, supported a deregulation of arginine metabolism within the urea cycle in Th1 NSCs (Fig. 6e). Since arginase is the urea cycle enzyme involved in the conversion of arginine to ornithine and urea (Fig. 6e), we hypothesized that the observed metabolic changes were depending on the defects of arginase activity. To verify this hypothesis, we measured the arginase enzymatic activity in cell extracts and assessed urea production by a colorimetric assay. Th1 NSCs showed a significant decrease in the arginase activity (Fig. 6c) and in the rate of urea production, compared to basal and Th2 NSCs (*p* ≤ 0.05 and *p* ≤ 0.01) (Fig. 6d).

Altogether, these findings suggest that inflammatory cytokine signalling causes a selective defect of arginase activity in Th1 NSCs.

### Expression of different arginase isoforms in NSCs

**Fig. 7:**
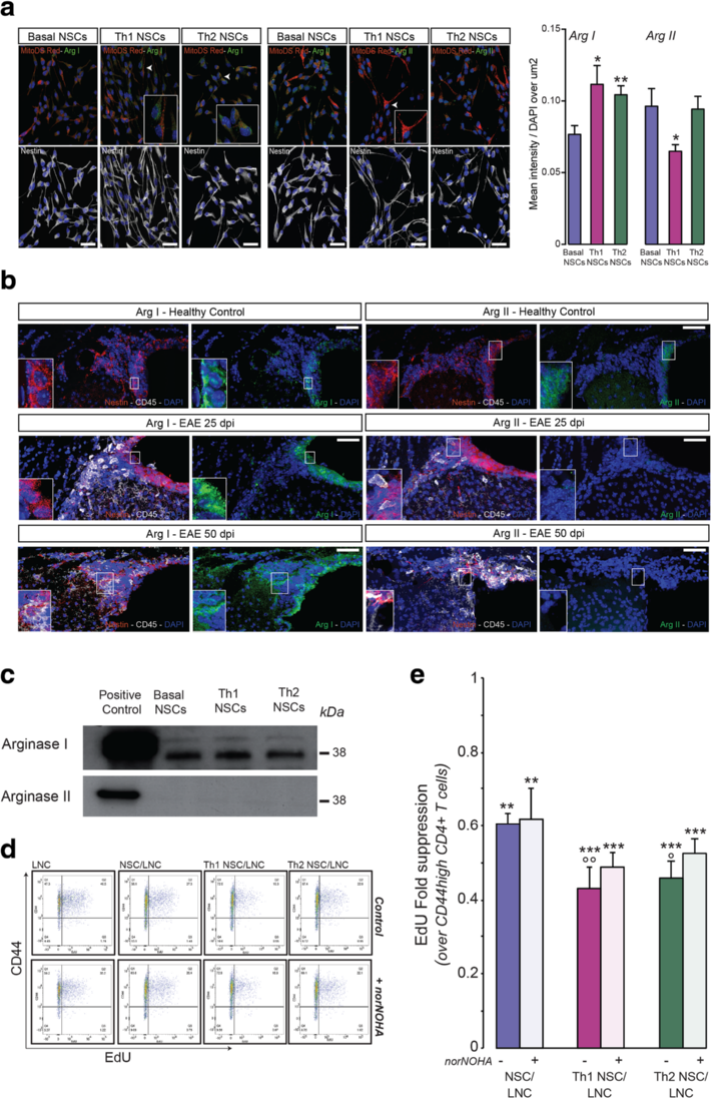
Cytokine-primed NSCs hinder LNC proliferation via a mechanism that is partly dependent on secreted arginase I. **a** Cytosolic arginase I (*green*) expression in Nestin (*white*) immunoreactive NSCs is increased upon priming with Th1 and Th2 cytokines (*arrowheads*). Arginase II (*green*) co-localizes mostly with red mitochondria (*arrows*), and it is reduced by priming with Th1 cytokines only. Mitochondria are in *red*, while nuclei are counterstained with DAPI (*blue*). Data are expressed as mean intensity (±SD) over total DAPI and have been collected out of *n* ≥ 3 independent experiments. *p ≤ 0.05 and **p ≤ 0.01 vs. basal NSCs. *Scale bars* 40 μm. **b** After EAE (25 and 50 days post immunization—dpi), arginase I expression is increased in Nestin^+^ SVZ-resident NSCs (*red*), compared to healthy controls (*left panels*). On the contrary, arginase II expression by Nestin^+^ SVZ-resident NSCs (*red*) is reduced in EAE mice compared to healthy controls (*right panels*). CD45-positive leukocytes are showed in *white*; nuclei are counterstained with DAPI (*blue*). *Scale bars* 50 μm. **c** Arginase I and arginase II protein expression in NSC supernatants detected by western blotting. Western blot analyses were performed starting from the same protein quantity (100 μg of total protein). This panel is representative of *n* ≥ 3 independent experiments. **d** Representative dot plots of EdU^+^CD44^high^ CD4^+^ T cells. **e** Relative fraction of proliferating EdU^+^ cells within CD44^high^CD4^+^ viable T cells. Data are expressed as mean EdU fold suppression (±SD) calculated dividing the percent of EdU^+^ cells in experimental samples over controls from *n* ≥ 3 independent experiments. Unpaired two-tailed *t*-test for comparisons between groups were applied; **p ≤ 0.01 and ***p ≤ 0.001 vs. LNC; °*p* ≤ 0.05 and °°*p* ≤ 0.01 vs. NSC/LNC

To further understand the regulation of arginase in cytokine-primed NSCs, we first investigated the expression of inducible (type I) and constitutive (type II) arginases in NSCs in vitro. We used immunofluorescence staining of Mito-DsRed-transduced Nestin^+^ NSCs to quantify the subcellular localization of cytosolic (type I) and mitochondrial (type II) arginase isoforms. We observed low basal expression of both arginases in NSCs and found that NSCs primed with Th1 cytokines had a significantly downregulated expression of arginase II, coupled with a significantly increased expression of arginase I (Fig. 7a).

As confirmation of these in vitro data, we interrogated a more physiological inflammatory context to examine the expression of both arginases by NSCs in vivo within brain germinal niches [40]*.* In mice with myelin oligodendrocyte glycoprotein (MOG)-induced experimental autoimmune encephalomyelitis (EAE), as model of Th1-like antigen-specific chronic inflammatory disease of the central nervous system [41], we found a downregulation of arginase II and concomitant upregulation of arginase I in Nestin^+^ NSCs of the SVZ at both 25 and 50 days after immunization vs. healthy controls (Fig. 7b).

These findings suggest that the previously observed decrease in the intracellular arginase activity in Th1 NSCs might well be ascribed to an imbalance between decreased arginase II expression (e.g. controlled at the post-translational level) and increased arginase I.

We then reasoned that (i) a shift in arginine metabolism and (ii) subsequent secretion of active arginases might be driving some of the immune modulatory properties of cytokine-primed NSCs.

To address the latter of these outstanding questions, we performed western blot analysis for both arginase I and II in NSC-conditioned media and found evidence of secreted arginase I, but not of arginase II (Fig. 7c). The secretion of arginase I might be an active secretion process by NSCs, instead of being a bystander effect of NSC death, as suggested by the comparable viability of basal, Th1 and Th2 NSCs (Additional file 5: Figure S3).

### Extracellular arginase I drives NSC modulation of T cell responses

To address the role of extracellular arginases in the immune modulatory effects of Th1 NSCs, we co-cultured basal, Th1, and Th2 NSCs with LNCs in the absence or in the presence of the potent, reversible, pan-arginase inhibitor N^ω^-hydroxy-nor-arginine (nor-NOHA) and measured EdU incorporation after 72 h in vitro.

Co-cultures with NSCs induced significant 40 % reduction of the fraction of CD44^high^EdU^+^ CD4^+^ T cells vs. LNCs (*p* ≤ 0.01). This effect was more striking in co-cultures with Th1 and Th2 NSCs, respectively (58 and 52 % reduction; *p* ≤ 0.01 or *p* ≤ 0.05 vs. NSC/LNC and *p* ≤ 0.001 vs. LNC). Pan-arginase inhibition by nor-NOHA had no effects on NSC/LNC but induced a 13 % rescue of the anti-proliferative properties of Th1 NSCs (*p* ≤ 0.01 vs. NSC/LNC + nor-NOHA) and a further 1.8-fold lower rescue of the anti-proliferative effects in Th2 NSC/LNC (*p* ≤ 0.05 vs. Th1 NSC/LNC + nor-NOHA) (Fig. 7d, e).

The fact that cytokine-primed NSCs were still in part capable to inhibit T cells proliferation in the presence of pan-arginase inhibition suggests that mechanisms other than extracellular arginase I or II contribute to the broad NSCs immune regulatory activities, as described elsewhere [3].

Overall, our findings suggest that deregulation of the arginine pathway and the release of arginase I contribute to part of the NSCs immune regulatory activities, especially in the case of NSCs primed with extracellular inflammatory signals.

## Discussion

Recent studies revealed that NSCs secrete neuroprotective and immune regulatory molecules able to mediate a cross talk with the surrounding tissues after transplantation and provide significant modulation of immune responses both in vitro and in vivo [16, 42]. While no final mechanisms or direct evidence of stem cell-to-immune system interaction is yet available, a number of studies are now focussing on the cellular signalling between grafted stem cells and endogenous target cells, with the aim of clarifying its physiological or circumstantial nature, and elucidating its molecular signature and therapeutic potential [15, 43, 44]. Whether and how cytokines produced in the CNS inflammatory or ischaemic environment in vivo modulate the NSCs immune regulatory activity is not unravelled yet but represents an intriguing possibility in developing more effective NSC-based therapeutic approaches [16]. Therefore, we reasoned that combined metabolomics-metabolic flux studies could provide useful insights into the NSC-immune cells crosstalk [45] elucidating the role of cytokines stimulation in the NSC metabolic reprogramming.

Previous evidence revealed that preconditioning (*priming*) of human NSCs by pro-inflammatory cytokines increased their cytotoxic activity towards human monocytic and lymphocytic cell lines via a TNF-α-mediated mechanism [14]. Here, we confirmed the existence of a crosstalk between NSCs and LNC/T cells, which is suggested by a premature downregulation of the activation marker CD44 on T cells and the failure of LNC to progress through the cell cycle and to proliferate. We also found that exposing NSCs to inflammatory Th1-like or Th2-like cytokines augmented the innate NSCs ability to repress T cells proliferation.

In the attempt to find a metabolic hypothesis for the reduced T cell proliferation promoted by Th1 or Th2 NSCs, we performed untargeted metabolomics by LC-MS/MS and GC-MS/MS, which covered a wide panel of both intracellular and extracellular metabolites belonging to the most important biochemical pathways. By high-throughput metabolomics, we found an alteration of intracellular arginine metabolism in NSCs, which was especially evident in Th1 NSCs and well correlated with the NSC anti-proliferative effect on T cells. We observed that the metabolism of arginine and proline was the most significant altered pathway for Th1 NSCs. This was supported by a significant decrease of arginase enzymatic activity and urea production as well as by the upregulation of other intracellular downstream arginine metabolites such as agmatine, N-acetylputrescine and 4-guanidinobutanoate.

Relative metabolite concentration changes are not sufficient to infer directionality of metabolic rates (fluxes) [46]. Thus, to investigate and validate changes in arginine metabolism, we incubated cells with U-^13^C_6_ L-arginine. This analysis confirmed the arginine pathway as the most relevant biochemical system affected by Th1-like (but not Th2-like) cytokines. The upregulation of arginine downstream metabolites such as agmatine, N-acetylputrescine and 4-guanidinobutanoate shown by untargeted metabolomics might mirror the significantly decreased amount of intracellular arginine—as such being predictive of faster arginine turnover—in Th1 NSCs. Changes in intracellular metabolic fluxes were best detected within 2 h of cytokine stimulation, while NSC inhibitory activities in NSCs/T cell co-cultures were observed at later times (48–72 h). We speculate the former changes to occur early after cytokine stimulation, while the latter ones to be better observed at later times as possibly caused by transcriptional or translational events controlled by changes in NPCs metabolic fluxes.

Arginase catalyzes the conversion of arginine to ornithine and urea [47]. While arginase II is constitutively expressed as the principal isoform in the nervous system and is localized in the mitochondria [48], arginase I is normally not expressed but induced in the cytosol. Arginase I expression in myeloid-derived suppressor cells (MDSCs) mediated their immunosuppressive activity in cancer patients [49] by depleting arginine in the tumour microenvironment and subsequently inhibiting T cell proliferation [49–51]. Arginase I secretion by mature myeloid cells in the tumour microenvironment inhibits antigen-specific T cell responses [52]. Likewise, overexpression of the extracellular arginase I contribute to the anti-inflammatory phenotype of phosphatase and tensin homolog (PTEN)-deficient myeloid cells [53].

We found that in vitro NSCs constitutively express arginase I, upregulate its expression upon priming with Th1 or Th2 cytokines and secrete it in tissue culture media. This is not surprising, as arginase I is not highly expressed in the healthy CNS [40, 54], but it is instead significantly upregulated in inflammatory conditions. As a matter of fact, sustained enhancement of arginase I expression has been well described during EAE within the spinal cord of both mice and rats [55]. However, this increase has been related so far to the prevailing M2 phenotype of local mononuclear phagocytes rather than being intrinsic to the CNS parenchyma [56]. Our findings suggest that arginase I is indeed upregulated in Nestin^+^ NSCs of the SVZ at both 25 and 50 dpi in EAE mice.

We also found that in vitro NSCs constitutively express arginase II and its expression is reduced by Th1 cytokines in vitro (while Th2 cytokines have no effect). Accordingly, in our in vivo settings, we found that arginase II is downregulated in the SVZ of EAE mice at chronic time points (i.e. 25 and 50 dpi). This is in line with previous data showing that arginase II gene expression rises in the spinal cord of EAE mice at the peak of disease, but it soon falls to levels below the baseline during recovery [57].

To understand whether changes in arginase activity in NSCs could be involved in their immune modulatory properties, we treated NSCs with the pan-arginase inhibitor nor-NOHA. We found that nor-NOHA had no effects on NSC/T cells but induced significant rescue of the anti-proliferative effects especially for Th1 NSCs in co-cultures with T cells in vitro. In the presence of nor-NOHA, Th1 NSCs—and to a 1.8-fold lower extent Th2 NSCs—no longer differed from control NSCs, thus suggesting that arginase inhibition partially rescues the anti-proliferative effects of NSCs. As arginase I—but not arginase II—was mainly present in the extracellular compartment, we speculated that nor-NOHA could mostly inhibit extracellular arginase I function in NSC/LNC co-cultures.

However, we cannot exclude that this pan-arginase inhibitor could also have an effect on the intracellular arginase II and therefore the observed rescue of the anti-proliferative activity observed for both Th1 and Th2 NSC/LNC may well be the sum of both the effects of the arginase inhibitor on intracellular arginase II and extracellular arginase I, as described [58].

We therefore hypothesized that defective intracellular arginase activity in cytokine-primed NSCs induces a rearrangement of intracellular arginine pool and that this effect together with the secretion of arginase I might reduce the pool of extracellular arginine, needed for proper T cell expansion.

Consistent with this hypothesis, we showed that the arginase I NSCs secretion could be partly responsible for the immune suppressive effects especially when NSCs *sense* the inflammatory environment (e.g., via cytokine receptors) [15].

The finding that nor-NOHA only partially rescued the anti-proliferative activity of cytokine-primed NSCs suggests that targeting the arginase activity in NSCs could provide a new way of interfering with NSCs function in vivo and that mechanisms other than extracellular arginase I would contribute to the broad NSCs immune regulatory activities [3].

## Conclusions

In conclusion, our work validates the use of metabolic profiling as a hypothesis-generating tool to unravel how stem cell-mediated mechanisms of tissue restoration are affected by local inflammatory responses and identifies arginase signalling as novel mechanism of adaptive NSC-to-immune system communication. While further studies are required to establish the absolute relevance of the NSC arginase-dependent immune modulatory mechanism, targeting arginase activity in NSCs could provide a new way of enhancing therapeutic immune-regulation.

Besides guiding our hypothesis on NSC immune-regulation on arginine pathway, this metabolomics approach offers a more broad and global profiling of NSCs under cytokines stimulation with other interesting pathways affected by Th1 pro-inflammatory cytokines that include phenylalanine, tyrosine and tryptophan metabolism, which are still under intense investigation. The very same technology might also be useful to investigate whether the crosstalk between NSCs and T cells occurs in a bi-directional way and if T cells alter the NSCs secretome.

Finally, we foresee that this high-throughput *omic* approach applied to the study of the NSCs phenotype might also provide promising therapeutic perspectives for inflammatory diseases where metabolic rearrangement plays a key role in disease progression.

## Additional files

Additional file 1: Figure S1. NSCs induce downregulation of the activation cell surface marker CD44 on purified CD4^+^ T cells. CD4^+^ T cells were purified by negative selection from total LNC, labelled with the vital dye CFSE, and cultured on CD3/CD28 coated mAb in the absence or the presence of NSCs, Th1 NSCs or Th2 NSCs for 72 h. Cells were surface stained with anti-CD4 and CD44 antibodies. Absolute fractions of CFSE^dim^CD44^low^ CD4^+^ T cells are depicted. Data are expressed as mean % (±SD) from *n* ≥ 3 independent experiments. **p* ≤ 0.05 and ***p* ≤ 0.01 vs. T cells. Additional file 2: Figure S2. NSCs reduce LNC death in NSC/LNC co-cultures. LNC were labelled with the vital dye CFSE, cultured on CD3/CD28 coated mAb in the absence or the presence of NSCs, Th1 NSCs or Th2 NSCs for 72 h. Cells were surface stained with anti-CD4, and TO-PRO3 Iodide was added before flow cytometry analysis to identify dead cells. Data represent absolute fractions of TO-PRO3^+^ CD4^+^ cells and are expressed as mean % (±SD) from *n* ≥ 3 independent experiments. ***p* ≤ 0.01 and ****p* ≤ 0.001 vs. LNC. Additional file 3: Table S1. NSC endometabolome (a) and exometabolome (b) detected by LC-MS/MS or GC-MS/MS. The endometabolome and exometabolome dataset comprise a total of 215 and 86 metabolites, respectively. Following log transformation and imputation with minimum observed values for each compound, Welch’s two-sample *t* tests were used to identify metabolites that differed significantly between experimental groups. A summary of the numbers of metabolites that achieved statistical significance (*p* ≤ 0.05), as well as those approaching significance (0.05 ≤ *p* ≤ 0.1), is reported. An estimate of the false discovery rate (*q* value) is calculated to take into account the multiple comparisons that normally occur in metabolomic-based studies. Additional file 4: Table S2. Metabolic pathways analysis of Th1 NSCs. This table is representative of the most significant pathways for Th1 NSCs compared to basal NSCs matched according to *p* values from pathway enrichment analysis and pathway impact values reported in Fig. 4. Additional file 5: Figure S3. Co-culturing with LNC and cytokine priming has no effects on NSC survival. LNC were labelled with the vital dye CFSE and co-cultured with NSC, Th1 NSCs or Th2 NSCs for 72 h as in Additional file 2: Fig. S2. Absolute fractions of CFSE^−^ TO-PRO3^+^ CD4^−^ cells, as an indicator of dead NSCs are expressed as mean % (±SD) from *n* ≥ 3 independent experiments.

## Abbreviations

BSTFA: Bistrimethyl-silyl-triflouroacetamide
CFSE: 5-(and-6)-carboxyfluorescein diacetate succinimidyl ester
CGM: Complete growth medium
DAPI: 4,6-diamidino-2-phenylindole
DCs: Dendritic cells
dpi: Days post immunization
EAE: Experimental autoimmune encephalomyelitis
EDTA: Ethylenediaminetetraacetic acid
EdU: 5-ethynyl-2′deoxyuridine
EGF: Epidermal growth factor
ESI: Electrospray ionization
FGF-2: Fibroblast growth factor
FT-ICR: Fourier transform ion cyclotron resonance
GC/MS: Gas chromatography/mass spectrometry
LC/MS: Liquid chromatography/mass spectrometry
LIT: Linear ion trap
LNC: Lymph node cells
MetPA: Metabolic pathway analysis
MOG: Myelin oligodendrocyte glycoprotein
MPCs: Myeloid precursor cells
nor-NOHA: N^ω^-hydroxy-nor-arginine
NSCs: Neural stem cells
OCT: Optimal cutting temperature
PBS: Phosphate-buffered saline
PFA: Paraformaldehyde
PLS-DA: Partial least squares discriminant analysis
PTEN: Phosphatase and tensin homolog
QA: Quality assurance
QC: Quality control
ROI: Region of interest
SVZ: Subventricular zone
TBST: Tris-buffered saline/Tween
TLR: Toll-like receptor
WB: Western blotting
