# Supplementary figures and images for: Metabolic determinants of the immune modulatory function of neural stem cells
Source: J Neuroinflammation. 2016 Sep 2;13(1):232. doi: 10.1186/s12974-016-0667-7 (PMC5009670; doi:10.1186/s12974-016-0667-7)

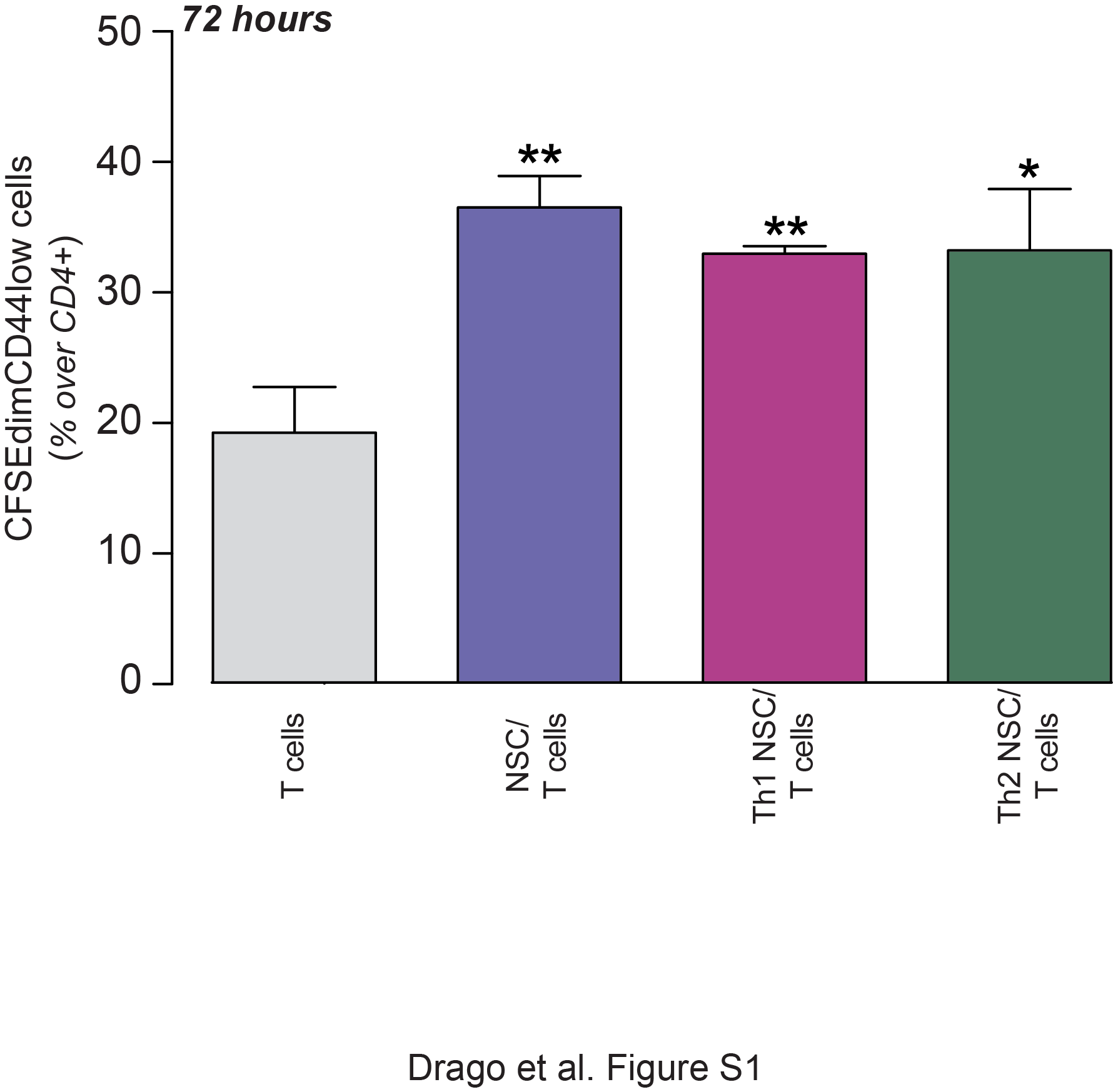

Supplement: Additional file 1: Figure S1. — NSCs induce downregulation of the activation cell surface marker CD44 on purified CD4+ T cells. CD4+ T cells were purified by negative selection from total LNC, labelled with the vital dye CFSE, and cultured on CD3/CD28 coated mAb in the absence or the presence of NSCs, Th1 NSCs or Th2 NSCs for 72 h. Cells were surface stained with anti-CD4 and CD44 antibodies. Absolute fractions of CFSEdimCD44low CD4+ T cells are depicted. Data are expressed as mean % (±SD) from n ≥ 3 independent experiments. *p ≤ 0.05 and **p ≤ 0.01 vs. T cells. [file 12974_2016_667_MOESM1_ESM.png]

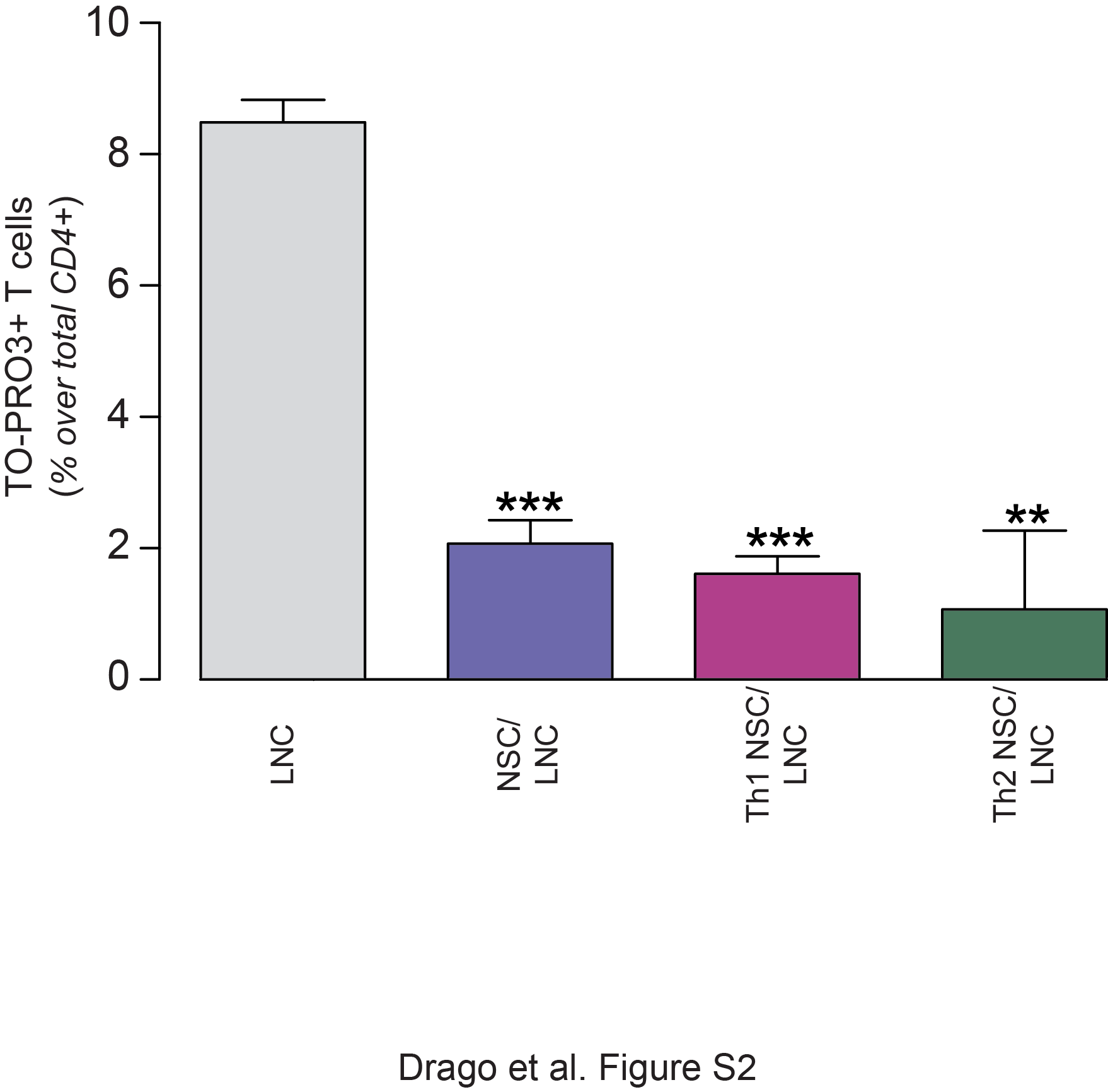

Supplement: Additional file 2: Figure S2. — NSCs reduce LNC death in NSC/LNC co-cultures. LNC were labelled with the vital dye CFSE, cultured on CD3/CD28 coated mAb in the absence or the presence of NSCs, Th1 NSCs or Th2 NSCs for 72 h. Cells were surface stained with anti-CD4, and TO-PRO3 Iodide was added before flow cytometry analysis to identify dead cells. Data represent absolute fractions of TO-PRO3+ CD4+ cells and are expressed as mean % (±SD) from n ≥ 3 independent experiments. **p ≤ 0.01 and ***p ≤ 0.001 vs. LNC. [file 12974_2016_667_MOESM2_ESM.png]

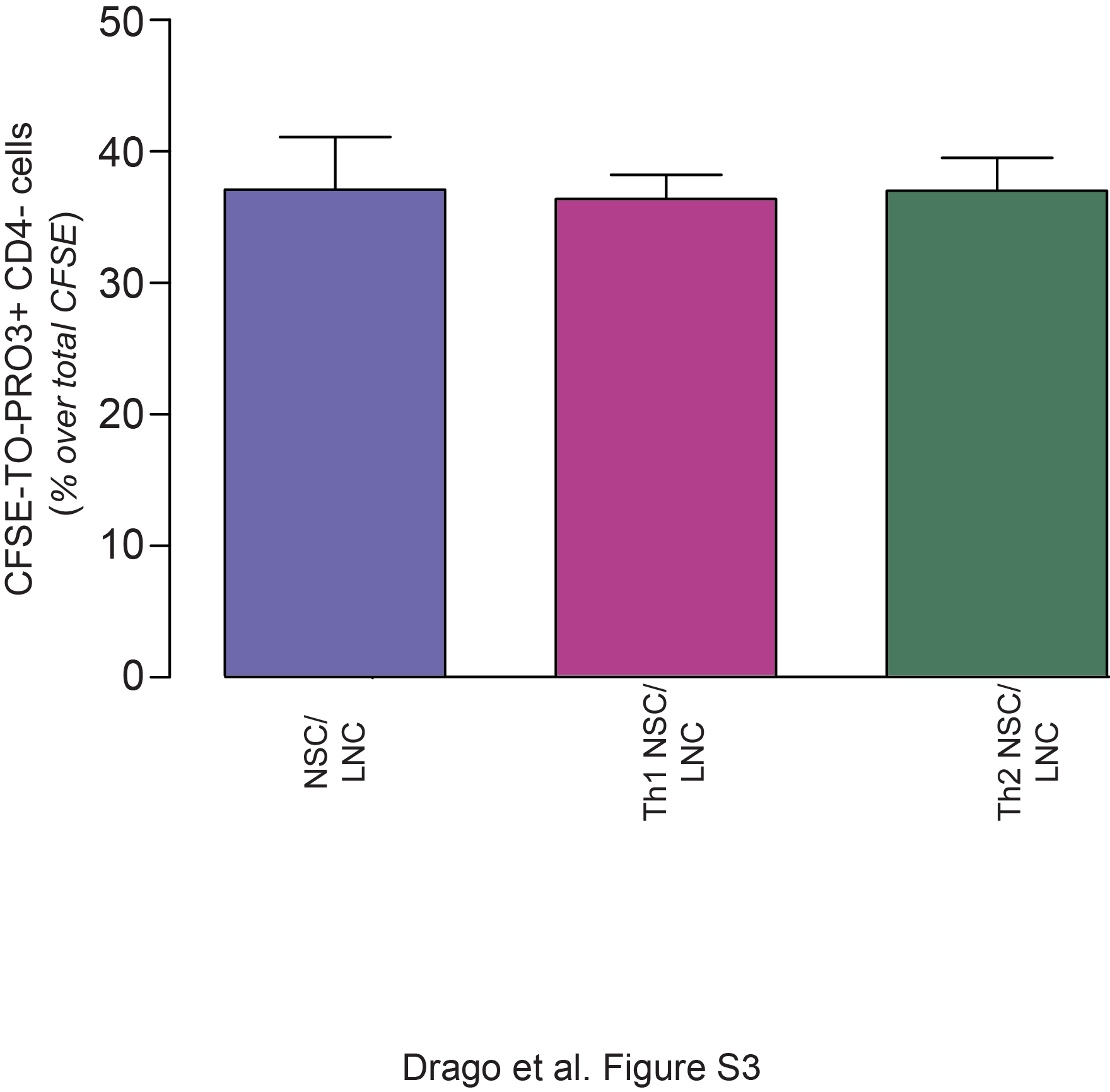

Supplement: Additional file 5: Figure S3. — Co-culturing with LNC and cytokine priming has no effects on NSC survival. LNC were labelled with the vital dye CFSE and co-cultured with NSC, Th1 NSCs or Th2 NSCs for 72 h as in Additional file 2: Fig. S2. Absolute fractions of CFSE− TO-PRO3+ CD4− cells, as an indicator of dead NSCs are expressed as mean % (±SD) from n ≥ 3 independent experiments. [file 12974_2016_667_MOESM5_ESM.png]
